# Stigma, culture, and mental health help-seeking among Black African immigrants in the United States: an integrative literature review

**DOI:** 10.1080/28324765.2026.2686466

**Published:** 2026-06-10

**Authors:** Ayuk Ngwi Tanyi Ashu

**Affiliations:** a College of Social Work, University of Kentucky

**Keywords:** Mental health stigma, Black African immigrants, help-seeking, culture and spirituality, intersectionality, social work practice

## Abstract

Stigma surrounding mental health continues to be a significant obstacle to seeking help and using services among Black African immigrant communities in the United States. Despite rapid population growth, Black African immigrants are frequently grouped with African Americans or omitted from mental health research, obscuring culturally specific experiences and needs. This integrative literature review synthesizes peer-reviewed research published between 2012 and 2025 to examine how stigma shapes mental health perceptions, help-seeking behaviors, and access to care among Black African immigrants. A systematic search was conducted across five databases: PsycINFO, APA PsycNET, PubMed, Infokat UKY, and Google Scholar. Of the 72 screened articles, only 22 met the inclusion criteria. The selected studies included qualitative, quantitative, integrative/scoping, conceptual/theoretical, and reports articles. Using an integrative synthesis approach, three main themes emerged: the cultural and spiritual understandings of mental illness; the influence of family and community norms on help-seeking behaviors; and structural and migration-related barriers in U.S. healthcare systems. Guided by Afrocentric and intersectionality frameworks, the review shows how stigma manifests at individual, interpersonal, and systemic levels, reinforced by religiosity, community expectations, immigration stressors, and structural racism. Across the studies, stigma consistently shaped perceptions of mental illness, delayed help-seeking, and reduced engagement with formal services. The findings underscore the importance of culturally responsive, community-based, and anti-racist social work and mental health interventions tailored to the varied experiences of Black African immigrants.

Mental health is defined as a condition of well-being in which a person recognises their own abilities, effectively manages daily stress, works productively, and gives back to their community (Herrman et al., 2005). However, perceptions of mental health and mental illness vary widely across cultural contexts and are shaped by spiritual worldviews, family norms, and historical experiences of marginalisation (Matsoele & Tadi, 2024). For many Black African communities, meanings attached to mental illness are shaped by cultural traditions, spiritual worldviews, family norms, and historical experiences of marginalisation. These meanings often intersect with stigma, creating substantial barriers to help-seeking and engagement with formal mental health services (Corrigan et al., 2012; Link & Phelan, 2001). Galderisi (2024) broadened this definition by characterising mental health as a positive sense of emotional and spiritual well-being, which appreciates culture, equity, social justice, interconnections, and personal dignity.

Stigma around mental illness remains a major obstacle to accessing treatment worldwide (Sartorius & Schulze, 2005, as cited in Dalky, 2011). It influences individuals through processes such as labelling, stereotyping, segregation, loss of status, and discrimination, leading to adverse effects on individuals, families, and communities (Link & Phelan, 2001; Rivera et al., 2021). For immigrants, stigma is further worsened by migration-related stress, racial discrimination, language difficulties, and limited culturally sensitive care (Iheduru-Anderson et al., 2025; Saasa et al., 2021). Specifically, Black African immigrants in the U.S. face additional hurdles due to the racialization of Blackness and the frequent neglect of their experiences in mental health research and services (Else-Quest et al., 2023; Omenka et al., 2020).

This review draws on Afrocentric and intersectionality frameworks to address these gaps. Afrocentric theory centres African worldviews, collectivism, spirituality, and communal responsibility as essential to understanding how mental illness is interpreted and managed within African diasporic communities (Borum, 2007; Hollingsworth & Phillips, 2017). Intersectionality highlights how race, gender, immigration status, class, and religion intersect to produce layered forms of stigma and structural disadvantage (Bubar et al., 2016; Else‑Quest et al., 2023). In this review, these frameworks guided the selection of studies that addressed cultural and structural dimensions of stigma, informed the coding and thematic synthesis, and shaped the interpretation of findings by emphasising how cultural meanings and structural inequities jointly influence help‑seeking among Black African immigrants.

## Types of stigmas

### Public stigma

Public stigma refers to the societal processes through which individuals with mental illness are labelled, stereotyped, and subjected to prejudice and discrimination (Corrigan et al., 2012; Rüsch et al., 2005). These reactions are shaped by cultural norms, collective beliefs, and social power structures that determine which attributes are viewed as acceptable or deviant. Within many Black African immigrant communities, public stigma is reinforced by cultural expectations surrounding family honour, fears of social exclusion, and community narratives that frame mental illness as a spiritual, moral, or supernatural issue (Gary, 2005; Tembo et al., 2025). Such beliefs often lead to gossip, social distancing, and warnings against associating with individuals perceived as mentally ill, which can result in isolation and diminished social support.

For Black African immigrants navigating life in the United States, public stigma is further intensified by racialization and structural inequities. Experiences of discrimination, marginalisation, and mistrust of healthcare systems shape how mental illness is perceived and discussed within immigrant communities (Omenka et al., 2020; Else‑Quest et al., 2023). These intersecting forces contribute to a heightened fear of being labelled, as mental illness may be seen as an additional vulnerability in a society where Blackness and immigrant status are already stigmatised.

### Self-stigma

Self‑stigma occurs when individuals internalise negative societal beliefs about mental illness and apply them to themselves, resulting in shame, reduced self‑esteem, and avoidance of treatment (Rüsch et al., 2005; Fox et al., 2018). Among Black African immigrants, self‑stigma is shaped not only by internalised cultural messages but also by migration‑related stressors, racial discrimination, and the pressure to maintain resilience in the face of acculturative challenges. Many immigrants report believing they should handle problems on their own, a perception reinforced by cultural norms that equate help‑seeking with weakness or personal failure (Saasa et al., 2021; Bassey & Zaka, 2024).

Self‑stigma is also influenced by what Fox et al. (2018) describe as experienced stigma, past encounters with stereotyping, prejudice, or discrimination. For Black African immigrants, these experiences may occur both within their communities and in broader U.S. society. When individuals internalise these experiences, they may minimise symptoms, delay seeking care, or rely exclusively on spiritual or non‑clinical coping strategies. Over time, self‑stigma can contribute to worsening mental health outcomes and reinforce cycles of silence and avoidance.

Recent national guidance reinforces the need to conceptualise immigrant mental health through updated psychological science. The *Psychological Science and Immigration Today* report emphasises that since 2012, research has increasingly integrated intersectionality, socioecological models, population health frameworks, and trauma-informed and decolonial approaches to understand immigrant well-being. The report notes that psychological research is gradually converging on key conclusions about immigrant health, particularly the role of structural conditions, discrimination, and policy contexts in shaping mental health outcomes (American Psychological Association, 2024, p. 6). These perspectives directly align with the present review’s focus on structural stigma and culturally grounded understandings of mental health among Black African immigrants.

## Population under study

This review focuses on Black African immigrants, defined as individuals born in sub‑Saharan African countries who currently reside in the United States. This population is analytically distinct from African Americans and Afro‑Caribbean immigrants due to differences in migration histories, cultural practices, languages, and social positioning. African immigrants represent one of the fastest‑growing immigrant groups in the United States, yet their mental health needs remain understudied and frequently obscured by data aggregation practices (Venters & Gany, 2011).

In 2018, approximately 2 million sub‑Saharan African immigrants lived in the United States, representing about 5% of the foreign‑born population (Saasa et al., 2021). When considering all Black immigrants, including those from the Caribbean, they account for roughly 1.3% of the total U.S. population (Tamir, 2022). Although relatively small in proportion, the Black immigrant population has grown rapidly over the past decades, with projections indicating that Black immigrants will constitute about one-third of the overall growth in the U.S. Black population by 2060 (Tamir, 2022). Despite this demographic expansion, African‑born populations remain statistically invisible in national mental health datasets, which often combine them with African Americans or broader “Black” or “foreign‑born” categories (Iheduru-Anderson et al., 2025).

Black African immigrants frequently arrive in the United States after experiencing significant pre‑migration stressors, including political violence, war, economic instability, and displacement (Omenka et al., 2020). Postmigration challenges, such as acculturative stress, racial discrimination, language barriers, and economic hardship, further elevate the risk of mental health concerns, including depression, anxiety, and post‑traumatic stress disorder (Botchway-Commey et al., 2024; Tembo et al., 2025). Despite these risks, Black African immigrants consistently demonstrate lower utilisation of mental health services compared to other racial and ethnic groups.

National data underscore this disparity. According to the National Survey on Drug Use and Health, 19.7% of Black and African American adults experienced a mental health condition in the past year, yet Black adults remain less likely to receive treatment compared to white or multiracial adults (Daniel et al., 2023). Additionally, research shows that Black adults are more likely than Asian adults to experience serious suicidal thoughts and are more likely to attempt or plan suicide compared to Asian and Native Hawaiian or Pacific Islander adults (Panchal et al., 2024). However, these statistics rarely disaggregate African‑born individuals, masking important cultural and structural factors that shape mental health experiences among Black African immigrants.

Stigma plays a central role in this underutilisation of services, yet much of the existing literature either conflates African immigrants with African Americans or treats Black immigrant populations as homogeneous. Iheduru-Anderson et al. (2025) emphasise that the African‑born population has more than doubled in recent decades but remains underrepresented in mental health research. This lack of specificity limits understanding of how culturally rooted beliefs, migration‑related stressors, and structural racism uniquely shape mental health stigma among Black African immigrants. By focusing specifically on this population, the present review seeks to address these gaps and contribute to a more culturally nuanced and empirically grounded understanding of mental health stigma within African immigrant communities.

This review intentionally uses the term Black African immigrants to highlight the intersection of race and migration status that shapes mental health stigma in the United States. While many primary studies refer to “African immigrants” or “sub-Saharan African immigrants,” these terms describe geographic origin but do not capture the racialization processes that African-born individuals experience upon arrival in the U.S. Demographic research identifies Black immigrants as a distinct population whose experiences differ from those of both African Americans and non-Black African immigrants, such as the North African immigrants (Tamir, 2022). Consistent with Afrocentric and intersectionality frameworks, the term Black African immigrants acknowledges that stigma is produced by the combined effects of cultural beliefs, migration histories, and anti-Black racism. This terminology does not imply homogeneity; rather, it makes explicit the racialized context in which African-born individuals navigate mental health and help-seeking in the United States.

## Problem identification

Although the Black African immigrant population in the United States has grown substantially over the past two decades, mental health research has not kept pace with this demographic shift. National mental health surveys have historically excluded African‑born populations or combined them with African Americans, masking important cultural, linguistic, and migration‑related differences (Iheduru-Anderson et al., 2025). This aggregation obscures the unique ways that stigma is formed, expressed, and sustained within Black African immigrant communities and limits the development of culturally responsive mental health interventions.

Black African immigrants face multiple, intersecting barriers to mental health care, including stigma, racism, acculturative stress, and limited access to culturally appropriate services. Cultural beliefs often frame mental illness through spiritual or moral lenses, contributing to fears of social exclusion, shame, and family dishonour (Iheduru-Anderson et al., 2025). These beliefs can discourage open conversations about mental health and delay help‑seeking. Additionally, structural obstacles like mistrust of medical systems and a lack of culturally competent providers contribute to the avoidance of formal mental health services.

Despite these challenges, the existing literature remains fragmented. Many studies conflate African immigrants with broader Black populations, while others focus narrowly on clinical outcomes without examining the cultural and structural processes that shape stigma. As a result, there is limited understanding of how culturally rooted beliefs, migration histories, and systemic inequities interact to influence mental health perceptions and help‑seeking among Black African immigrants. This lack of specificity limits the field’s ability to design culturally grounded, community‑driven, and responsive interventions for African‑born populations.

Given these gaps, a comprehensive synthesis of the literature is needed to clarify how stigma is conceptualised, experienced, and maintained within Black African immigrant communities. This integrative review addresses this need by examining the multilevel factors that shape mental health stigma and by identifying opportunities for culturally responsive and anti‑racist mental health practice.

## Theoretical framework

This review draws on Afrocentric and intersectionality frameworks to provide a culturally grounded and structurally informed lens for understanding mental health stigma among Black African immigrants in the United States. These frameworks informed the review by guiding the selection of studies that addressed cultural, structural, and identity‑based dimensions of stigma. The frameworks also shaped the thematic synthesis by highlighting how race, migration status, spirituality, and community norms intersect to influence help‑seeking.

### Afrocentric framework

Afrocentric theory centres African worldviews, cultural values, and historical experiences as essential to understanding the lives of people of African descent (Hollingsworth & Phillips, 2017). In the context of African immigrants, core principles of Afrocentricity, such as collectivism, spirituality, interconnectedness, respect for elders, and communal responsibility, inform how mental illness is interpreted and addressed within African diasporic communities. For many Black African immigrants, emotional distress is often understood through spiritual or relational frameworks rather than biomedical ones, influencing both perceptions of mental illness and pathways to care (Borum, 2007; Jones et al., 2007).

Although Afrocentric scholarship has primarily developed within African American contexts, its principles are adaptable to African immigrant communities when applied with cultural specificity (Gilbert et al., 2009). An Afrocentric approach challenges deficit-focused narratives and highlights cultural assets, resilience, and traditional knowledge systems that influence how mental health issues and stigma are addressed. Afrocentricity addressed cultural meanings of mental illness, spirituality, family and community norms, and traditional healing practices. It highlights themes related to cultural identity, communal expectations, and the role of spirituality in shaping stigma and help‑seeking.

### Intersectionality framework

Intersectionality provides a critical lens for examining how multiple social identities and power structures interact to shape lived experiences (Bubar et al., 2016; Simon et al., 2022). For Black African immigrants, stigma is not solely a cultural phenomenon; it emerges at the intersection of race, immigration status, gender, class, religion, and language (Else-Quest et al., 2023; Oexle & Corrigan, 2018). Factors such as structural racism, xenophobia, and immigration-related marginalisation interact with cultural stigma to form multiple, layered barriers to accessing mental health care (Bamgbose Pederson et al., 2022; Berger et al., 2018).

Intersectionality informed inclusion of studies that addressed structural racism, xenophobia, acculturative stress, and the racialization of Blackness. It also shaped the thematic synthesis by drawing attention to how cultural stigma interacts with systemic inequities, such as mistrust of healthcare institutions, limited culturally responsive services, and experiences of racial discrimination to influence help‑seeking behaviours.

Together, Afrocentric and intersectionality frameworks conceptualise stigma as both culturally embedded and structurally reinforced. This integrated approach is particularly useful for informing culturally responsive, anti-racist, and community-based mental health and social work interventions (Hunting and Grace, 2015; Rivera et al., 2021).

## Methodology

### Purpose and design

This study employs an integrative literature review design to synthesise empirical, theoretical, and reports literature on mental health stigma among Black African immigrants in the United States. An integrative approach was selected because it allows for the inclusion of diverse methodologies, qualitative, quantitative, mixed‑methods, conceptual, and review studies, thereby supporting a comprehensive understanding of how stigma is shaped by cultural, spiritual, and structural factors.

### Search strategy

A systematic search was conducted across five databases: PsycINFO, APA PsycNET, PubMed, Infokat UKY, and Google Scholar. Search terms included combinations of: mental health stigma, Black African immigrants, African migrants, help‑seeking, culture, spirituality, and intersectionality. The search was limited to English‑language publications from 2012 to 2025. Reference lists of included studies were also reviewed to identify additional sources.

### Inclusion and exclusion criteria

The database search identified 72 records. These were initially screened for relevance based on titles and abstracts. All studies that appeared potentially suitable underwent a full-text review. Inclusion criteria mandated that studies focus on Black African or African immigrant populations, explore mental health stigma, perceptions of mental illness, or help-seeking behaviours, and employ qualitative, quantitative, mixed-methods, conceptual, or review methodologies. Exclusions applied to studies that did not distinguish African immigrants from other Black groups, focused solely on clinical outcomes without addressing stigma, or lacked sufficient information on mental health or stigma.

## Result

**Figure 1.**
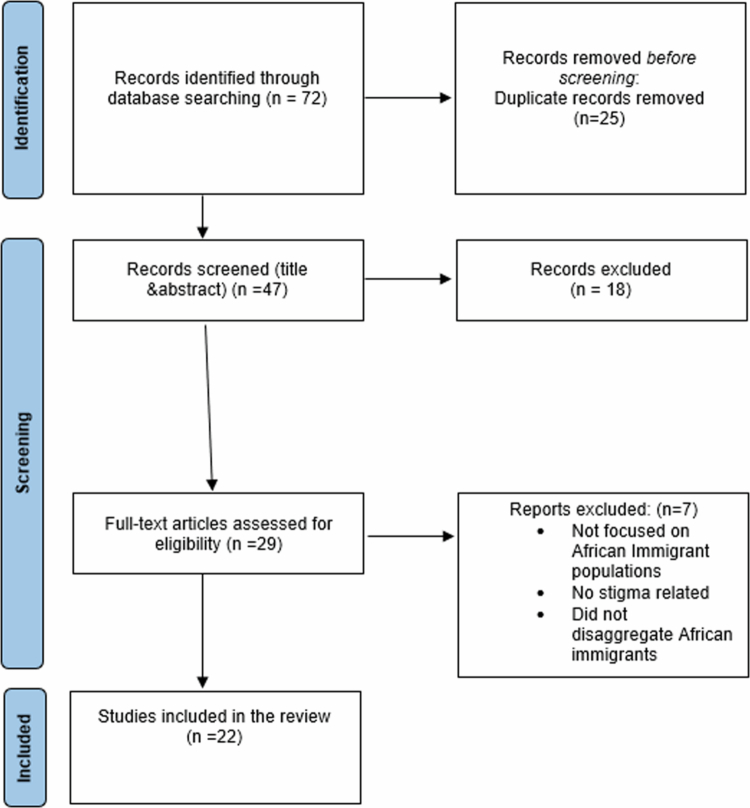
PRISMA flow diagram of data search.

The PRISMA flowchart below illustrates the article screening process depicted in Figure 1. The overall screening yielded 72 articles, of which 25 were duplicates. After reviewing their abstracts, 18 articles were excluded because they did not meet the inclusion criteria, such as studies focusing on other minority groups like Latinos and the Hispanic population. Of the remaining articles, 29 were considered; however, 7 were excluded for failing additional criteria, including the absence of stigma-related content or failure to disaggregate data for African immigrants. Ultimately, 22 articles were incorporated into the integrative synthesis review. A PRISMA‑style flow diagram summarises the identification, screening, exclusion, and inclusion process. Screening was conducted by the author. The use of a single reviewer is acknowledged as a methodological limitation.

### Data analysis

The articles were reviewed multiple times and analysed using thematic synthesis. The process included open coding of all studies to uncover how stigma was defined, experienced, and connected to cultural, spiritual, familial, and structural factors. Codes were grouped into categories via an iterative process guided by Afrocentric and intersectionality frameworks. Themes were developed inductively, with these frameworks highlighting cultural meanings and power dynamics. The themes were reviewed and refined for clarity and coherence across the studies. Key characteristics of the 22 studies, such as purpose, design, sample, and main findings, were extracted and summarised. These details are compiled in Table 1, offering an overview of the evidence supporting this review.

**Table 1.** Data extraction table.

Author(s) & Year	Purpose	Design/Method	Sample/Population	Key Findings	Relevance
Bamgbose Pederson et al. (2022)	Explore stigma among African immigrant perinatal women	Qualitative brief report	African immigrant pregnant/postpartum women (U.S.)	Stigma, shaped by cultural norms and the fear of judgement, limits help‑seeking.	Highlights gendered and cultural stigma barriers
Bassey and Zaka (2024)	Identify barriers to service use	Systematic review	African immigrants (UK)	Stigma, mistrust, and cultural beliefs reduce service utilisation.	Strong evidence on systemic barriers
Botchway-Commey et al. (2024).	Review help-seeking behaviours	Systematic review	African migrants	Migration stress and cultural beliefs shape help‑seeking patterns.	Core evidence for stigma impact
Dein (2018)	Advocate for religion in care	Commentary	Psychiatric care	Spiritual interpretations both hinder and support coping.	Cultural/spiritual competence
Galvin et al. (2023)	Explore traditional beliefs	Qualitative	South African healers	Mental illness attributed to spiritual causes; stigma reinforced.	Alternative care systems
Ganga and Kutty (2012)	Examine religion & mental health	Quantitative	Youth	Faith communities provide support and shape beliefs about mental health.	Protective factors
Gary (2005)	Examine stigma in minorities	Literature review	Ethnic minorities	Stigma is a major barrier to care among minority groups.	Foundational stigma research
Iheduru-Anderson et al. (2025)	Review service utilisation	Integrative review	African immigrants (U.S.)	African immigrants underrepresented; stigma reduces service use.	Highly relevant core study
Knaak et al. (2017)	Address stigma in healthcare	Review	Healthcare systems	Stigma in healthcare reduces access and quality of care.	System-level barrier
Matsoele and Tadi (2024)	Examine mental health literacy	Quantitative	South African communities	Mental illness framed through spiritual worldviews.	Education gap
Okafor et al. (2022).	Role of traditional beliefs	Quantitative	Nigerian women	Mental illness is seen as a spiritual attack; stigma reduces help‑seeking.	Cultural explanatory models
Omenka et al. (2020)	Review healthcare experiences	Scoping review	African immigrants (U.S.)	Mistrust of U.S. healthcare systems; racism shapes experiences.	Service delivery gaps
Panchal et al. (2024)	Examine disparities	Survey report	U.S. racial groups	Black adults report higher suicidal ideation and disparities in care.	Policy relevance
Pederson et al. (2023)	Religiosity & stigma	Quantitative	Black populations	Higher religiosity is associated with increased stigma.	Complex cultural role
Pederson et al. (2022)	Ethnic identity & stigma	Quantitative	Black adults	Strong identity linked to stigma attitudes	Identity factor
Pedersen and Paves (2014)	Young adults (U.S.)	Quantitative	Young Adults	Public vs. personal stigma	Personal stigma stronger predictor of avoidance.
Rivera et al. (2021)	Review stigma interventions	Narrative review	African Americans	Limited culturally tailored interventions	Reviews interventions targeting stigma in Black communities.
Rüsch et al. (2005)	Review stigma concepts	Literature review	General	Defines stigma processes and consequences.	Foundational
Saasa et al. (2021)	Examine service utilisation	Quantitative	African immigrants (U.S.)	Underutilisation due to stigma and access	Core empirical evidence
Tembo et al. (2025)	Explore stigma & help-seeking	Qualitative	African migrants (Australia)	Stigma is shaped by spirituality and social exclusion.	Cross-national relevance
Venters and Gany (2011)	Review African immigrant health	Literature review	African immigrants	Structural barriers and stigma reduce access to care.	Broad health context
Wu et al. (2025)	Examine stigma & utilisation	Quantitative	Black adults	Stigma moderates service use	Strong recent empirical evidence

### Ethical considerations

As a secondary analysis of published literature, this review did not involve human subjects and did not require institutional review board approval. Ethical standards were upheld by accurately representing sources, using culturally respectful language, and interpreting findings with sensitivity to the experiences of Black African immigrant communities.

## Discussion

The findings of this integrative review show that mental health stigma among Black African immigrants operates across individual, interpersonal, and structural levels (Knaak et al., 2017; Rüsch et al., 2005), shaped by cultural beliefs, spiritual interpretations, family expectations, migration‑related stressors, and systemic racism. Although the primary focus of this review is the U.S. context, several included studies were conducted in other Western or African settings. These studies were retained because they offer culturally relevant insights into stigma processes, particularly spiritual attributions of mental illness, communal norms, and migration‑related challenges, that parallel the experiences of African immigrants in the United States.

### Cultural and spiritual interpretation

Across the included studies, spirituality emerged as a central influence on how mental illness is understood and addressed. However, the role of spirituality was not uniform. Several qualitative studies described spirituality as reinforcing stigma by framing mental illness as a moral failing, spiritual attack, or consequence of ancestral displeasure (Okafor et al., 2022; Tembo et al., 2025). In contrast, other studies highlighted spirituality as a source of resilience, community support, and meaning‑making that facilitated coping and reduced isolation (Dein, 2018; Ganga & Kutty, 2012). This divergence suggests that spirituality functions as both a risk and protective factor, depending on denominational beliefs, leadership attitudes, and community norms.

### Family and community norms

Family and community dynamics also played a complex role. Public stigma, expressed through gossip, social distancing, and concerns about family honour, was consistently identified as a barrier to help‑seeking (Gary, 2005; Tembo et al., 2025). Nevertheless, some studies noted that families also acted as protective buffers, offering emotional support and encouraging alternative coping strategies such as prayer or traditional healing. These findings indicate that family systems can simultaneously perpetuate and mitigate stigma, underscoring the need for interventions that engage families as partners rather than viewing them solely as barriers (Rüsch et al., 2005; Fox et al., 2018; Saasa et al., 2021).

### Migration-related barriers

Differences across gender, migration status, and region further complicate the stigma landscape. Several studies found that women were more likely to internalise stigma and experience shame, whereas men were more likely to avoid help‑seeking due to norms of emotional stoicism and expectations of strength (Saasa et al., 2021; Bassey & Zaka, 2024; Bamgbose Pederson et al., 2022). First‑generation immigrants tended to endorse stronger spiritual or supernatural explanations for mental illness than second‑generation individuals, reflecting the influence of acculturation on stigma beliefs. Regionally, immigrants from countries with strong traditional healing systems were more likely to attribute mental illness to spiritual causes, whereas immigrants from more urbanised or Western‑influenced African contexts reported greater openness to biomedical explanations (Matsoele & Tadi, 2024; Pedersen & Paves, 2014).

### Structural racism

Structural racism and migration‑related stressors further intensified stigma. Experiences of discrimination, language barriers, and mistrust of U.S. healthcare systems discouraged engagement with mental health services even when symptoms were severe (Omenka et al., 2020; Iheduru‑Anderson et al., 2025). Some studies described mental illness as an additional threat to social survival in a context where Blackness and immigrant status are already stigmatised (Else-Quest et al., 2023). These findings highlight the need to conceptualise stigma not only as a cultural phenomenon but also as a structural one shaped by racialized power dynamics.

Methodological differences across studies also revealed important patterns. Quantitative studies consistently documented low rates of mental health service utilisation among African immigrants, but they often lacked the cultural nuance needed to explain why these patterns occur. Qualitative studies, in contrast, provided rich insight into the cultural, spiritual, and structural mechanisms underlying stigma, including fears of social exclusion, mistrust of healthcare systems, and concerns about racial discrimination. Together, these findings suggest that quantitative prevalence data alone cannot capture the complexity of stigma among Black African immigrants; mixed‑methods and qualitative approaches remain essential for understanding the lived experiences that shape help‑seeking.

Overall, the literature reveals both convergence and divergence in how stigma is experienced and expressed. While cultural and spiritual beliefs consistently shape perceptions of mental illness, the direction and intensity of their influence vary across gender, migration histories, and community contexts. The interplay between cultural stigma and structural racism remains a critical area for further research, particularly given the limited number of studies that explicitly examine these intersections.

## Clinical implications for social work and mental health practice

The findings of this review highlight several implications for social work and mental health practice that are both culturally responsive and grounded in the evidence synthesised across the included studies. The APA’s 2024 Task Force underscores that effective clinical practice with immigrant communities requires a diverse, culturally competent workforce, integration of wraparound services, and care delivered in accessible, community-based settings. The report highlights the need for clinicians to adopt cultural humility and to recognise how immigration policy, discrimination, and legal precarity shape mental health trajectories (American Psychological Association, 2024, pp. 28–32). These recommendations reinforce the implications of this review, particularly the need for clinicians serving Black African immigrants to address migration-related stressors, stigma, and structural barriers as part of routine mental health care. Moreover, while community engagement, faith-based partnerships, and anti-racist practice remain essential, the recommendations below are closely linked to the reviewed literature.

First, several studies have emphasised the central role of spirituality in shaping perceptions of mental health among Black African immigrants (Okafor et al., 2022; Tembo et al., 2025). Given this, practitioners should collaborate with faith leaders and spiritual communities not only to reduce stigma but also to leverage their influence as trusted sources of support. Evidence from qualitative studies suggests that when faith leaders receive psychoeducation and partner with mental health providers, community members demonstrate greater openness to discussing mental health concerns. Wu et al. (2025) call for future research and interventions to adopt nuanced approaches that address both mental illness and non-pathological emotional experiences, tailored to the specific needs of Black adults.

Practitioners need to tackle structural racism and power disparities in mental health systems. Providing training in cultural humility, anti-racism, and intersectionality is crucial for better engagement with Black African immigrant clients. Using community outreach and culturally adapted screening tools can help improve access to care. Additionally, empowering individuals and sharing personal stories to reduce stigma at both national and local levels may be especially effective in changing public perceptions and increasing the use of mental health services (Wu et al., 2025). Ongoing research, especially in U.S. contexts, continues to be crucial for understanding how migration, racism, and transnational identity influence stigma, and it helps develop effective, culturally responsive mental health interventions.

Second, the review shows that family and community norms significantly influence help‑seeking behaviours. Studies documenting the impact of public stigma, gossip, and concerns about family honour (Gary, 2005; Tembo et al., 2025) indicate that interventions must extend beyond individuals to include family‑centred and community‑centred approaches. Programs that incorporate family dialogue, community forums, or culturally adapted psychoeducation have been shown to reduce stigma and increase awareness of mental health symptoms.

Third, structural racism and mistrust of healthcare systems were consistently identified as barriers to service utilisation (Iheduru-Anderson et al., 2025; Omenka et al., 2020). This underscores the need for anti‑racist clinical practice, including culturally responsive screening tools, trauma‑informed engagement strategies, and workforce training in cultural humility. Evidence from the reviewed studies suggests that when providers demonstrate cultural attunement and acknowledge systemic inequities, African immigrant clients report greater trust and willingness to engage in care.

Finally, the divergence between qualitative and quantitative findings highlights the importance of integrating culturally grounded assessment methods. Quantitative studies documented low service utilisation, while qualitative studies illuminated the cultural and structural mechanisms underlying these patterns. Practitioners should therefore incorporate narrative‑based assessments, community consultations, and culturally adapted interventions to better align services with the lived experiences of Black African immigrants.

The American Psychological Association (2024) report calls for research approaches that are innovative, decolonised, and community-engaged, emphasising that immigrant populations must be involved in shaping research questions, methods, and interpretations. The Task Force recommends expanding community-based participatory research and prioritising studies that examine how policy environments, discrimination, and social determinants of health influence immigrant mental health. These recommendations align with the gaps identified in this review, particularly the need for research that centres Black African immigrants' lived experiences, captures within-group diversity, and examines structural stigma as a determinant of mental health.

### Limitations of the study

This review has certain limitations that should be recognised to interpret its findings properly. First, the search was restricted to English-language publications, potentially overlooking relevant studies published in other languages commonly spoken across Africa. This creates a potential language bias that could narrow the cultural diversity of the evidence base.

Second, publication bias may exist because the review focused primarily on peer-reviewed literature and did not include community-based reports, unpublished theses, or studies not indexed that discuss mental health stigma among African immigrants.

Third, although a basic quality appraisal was conducted, the methodological heterogeneity of the included studies, ranging from qualitative interviews to conceptual papers and systematic reviews, limits the ability to compare findings across designs. Some studies lacked methodological transparency, small sample sizes were common, and few studies explicitly examined intersectional or structural dimensions of stigma.

Fourth, the review process itself has limitations. The screening and coding were primarily performed by the author. The lack of multiple independent reviewers could lead to bias in selection or interpretation. Although efforts were made to improve rigour through iterative coding and framework-guided synthesis, future reviews could be strengthened by involving multiple coders and implementing formal inter-rater reliability procedures.

Although the review mainly focused on the U.S. context, some of the included studies were conducted in non-U.S. settings. These were retained for their cultural relevance, but their inclusion may limit the extent to which the findings apply to African immigrants in the U.S. Future research should focus on U.S.-based, disaggregated, and longitudinal studies to better understand stigma processes among this group.

## Conclusion

This integrative review demonstrates that mental health stigma among Black African immigrants is shaped by intersecting cultural, spiritual, familial, and structural forces that operate across multiple levels. While spirituality, family dynamics, and community norms strongly influence perceptions of mental illness, structural racism, migration‑related stressors, and mistrust of healthcare systems further compound stigma and reduce engagement with mental health services. The synthesis also reveals important variations across gender, migration histories, and regional backgrounds, underscoring the heterogeneity within African immigrant communities.

Several knowledge gaps emerged across the reviewed literature. First, few studies disaggregate African‑born individuals from African Americans or other Black immigrant groups, limiting the ability to identify culturally specific stigma processes. Second, there is a lack of research examining how structural racism and immigration policy intersect with cultural stigma to shape help‑seeking. Third, quantitative studies often document low service utilisation without exploring the cultural and structural mechanisms underlying these patterns, while qualitative studies provide depth but remain geographically limited. Finally, very few studies examine second‑generation African immigrants or compare stigma experiences across generations.

Addressing these gaps requires more detailed, U.S.-focused, long-term research, greater attention to intersectional and structural influences, and the creation of culturally sensitive, community-involved interventions. By pinpointing these gaps and integrating existing evidence, this review offers a more refined understanding of mental health stigma among Black African immigrants. It suggests ways to promote culturally equipped and anti-racist mental health practices.

These findings reinforce the updated national guidance from the American Psychological Association (2024), which calls for immigrant-centred, culturally responsive, and structurally informed approaches to psychological research and practice.

## Data Availability

Data sharing does not apply to this article as no new data were created or analysed.

## Impact statement

This is an integrative review paper. No human subjects were recruited, and IRB approval was not required.
